# *In vitro* study of probiotic *Lactobacillus helveticus*: Antibacterial effects on *Porphyromonas gingivalis*

**DOI:** 10.1371/journal.pone.0329497

**Published:** 2025-08-08

**Authors:** Husna Hazirah Bakri, Syarifah Nur Syed Abdul Rahman, Adyani Azizah Abd Halim, Nor Adinar Baharuddin, Hasnah Begum Said Gulam Khan, Zamirah Zainal Abidin, Mohd Hafiz Arzmi, Elly Munadziroh, Wan Himratul Aznita Wan Harun

**Affiliations:** 1 Department of Oral & Craniofacial Sciences, Faculty of Dentistry, Universiti Malaya, Kuala Lumpur, Malaysia; 2 Department of Restorative Dentistry, Faculty of Dentistry, Universiti Malaya, Kuala Lumpur, Malaysia; 3 Centre of Preclinical Science Studies, Faculty of Dentistry, UiTM Sg Buloh Campus, Selangor, Malaysia; 4 Department of Craniofacial Diagnostics and Biosciences, Faculty of Dentistry, Universiti Kebangsaan Malaysia, Kuala Lumpur, Malaysia; 5 Kulliyyah of Dentistry, International Islamic University Malaysia Kuantan, Pahang, Malaysia; 6 Department of Dental Material, Faculty of Dental Medicine, Universitas Airlangga, Surabaya, Indonesia; University of Pennsylvania, UNITED STATES OF AMERICA

## Abstract

Probiotics are gaining attention for their benefits as a supplement to improve oral health. This study aimed to evaluate the antibacterial effect of the probiotic *Lactobacillus helveticus* against *Porphyromonas gingivalis*, a significant pathogen in periodontal diseases. Antibacterial susceptibility was assessed using the well diffusion assay, with 0.12% chlorhexidine (CHX) served as the positive control. Biofilm biomass was evaluated using crystal violet staining. Cell viability in *P. gingivalis* treated with *L. helveticus* was determined using the LIVE/DEAD Baclight bacterial assay via fluorescence microscopy. Ultra-morphological alterations in these cells were further examined using Field Emission Scanning Electron Microscopy. The results indicated that *L. helveticus* significantly reduced the growth of *P. gingivalis*. The highest concentration of 10^9^ cells/mL achieved the most substantial inhibition in the well diffusion assay, followed by concentrations of 10^8^ cells/mL and 10^7^ cells/mL, which demonstrated a clear dose-dependent response. Furthermore, biofilms of *P. gingivalis* treated with *L. helveticus* exhibited a notable biomass reduction of up to 85% at the highest concentrations. LIVE/DEAD staining confirmed a decreased in cell viability among the treated populations, while FESEM analysis revealed morphological disruptions in *P. gingivalis* cells treated with *L. helveticus*. These findings suggest that *L. helveticus* has a potent antibacterial effect against *P. gingivalis*, highlighting the need for further research to identify the optimal probiotic strategies that could enhance periodontal health.

## 1. Introduction

Periodontitis is one of the most common inflammatory diseases affecting all age groups, but it has a higher prevalence in the elderly population [1]. Periodontitis has a prevalence rate of about 50% worldwide and most commonly occurs in adulthood. It is defined as a chronic inflammatory disease of the supporting tissues of the teeth of infectious origin [2]. The supportive tissues of the teeth that are affected by inflammation include the cementum, periodontal ligament, alveolar bone and gingiva surrounding the tooth [3,4]. Inflammation in these areas not only contributes to tooth loss but has also been associated with various systemic diseases, including diabetes, atherosclerosis, rheumatoid arthritis, Alzheimer’s disease, gastrointestinal disorders, and low birth weight in newborns [5]. Periodontitis is characterised by inflammation of the gingiva, which is associated to the buildup of dental plaque. This condition may progress to include inflammation of the bone and periodontal ligament, resulting in the formation of periodontal pockets, a key feature of the disease [6].

Periodontitis is characterised as a dysbiotic disease marked by a shift in the subgingival microbial communities within periodontal pockets, from predominantly Gram-positive aerobic bacteria to a dominance of Gram-negative anaerobes. Notably, the “red-complex” bacteria, which includes *Porphyromonas gingivalis*, *Tannerella forsythia*, and *Treponema denticola,* are significant contributors to this dysbiosis [7]. Among these, *P. gingivalis* is recognised as a primary causative agent closely associated with the development of periodontitis [8]. *P. gingivalis* is an obligate anaerobe that thrives in low-oxygen environments and has evolved various mechanisms that allow it to colonise beneath the gingiva, evade the host immune response, and cause damage to oral tissues. Its pathogenicity is enhanced by several virulence factors, including fimbriae, haemolysin, haemagglutinin, capsules, outer membrane vesicles, lipopolysaccharides, and gingipains. Furthermore, *P. gingivalis* has been shown to form biofilms, which can protect it from host immune responses and enhance its ability to persist within the oral environment [1]. Fimbriae are considered a critical factor in the onset of periodontitis. These surface structures consist of fimbrillin subunits [9], and are essential for various processes, including biofilm formation, auto-aggregation, co-aggregation with other oral bacteria, adhesion to host molecules, and invasion of host cells [10]. These invasion mechanisms contribute to the resorption of alveolar bone, the destruction of surrounding periodontal tissues, and an increased risk of developing systemic diseases associated with periodontitis [11].

Lactic acid bacteria (LAB) are non-spore-forming, Gram-positive, non-respiratory probiotics that generate lactic acid through the fermentation of carbohydrates. *Lactobacillus* species, widely recognised as probiotics and integral members of the human microflora across various sites, including the oral cavity, have gained increasing interest for their ability to inhibit the growth of numerous pathogens [12]. Recently, probiotics have garnered increasing attention for their potential benefits in improving oral health. Numerous studies have demonstrated the ability of probiotics to inhibit oral pathogens, either through live cultures or cell-free supernatants [13,14]. Among these, *L. helveticus*, one of the several species of lactic acid bacteria, has demonstrated several probiotic properties in previous *in vitro* studies, such as resilience in the gastrointestinal tract, adhesion to epithelial cells, and the capability to antagonize pathogenic microorganisms [15,16]. Apart from that, a previous study has reported that *L. helveticus* has been shown to reduce the increase of pro-inflammatory cytokine expression induced by *P. gingivalis* stimulation in gingival epithelial cells [17]. The antibacterial properties of probiotics are attributed to their production of organic acids, their ability to adhere to pathogens, and their capacity to decrease bacterial adherence. Furthermore, studies have shown that Lactobacilli produce various bactericidal compounds [18]. Evidences suggest that the antibacterial activity of *Lactobacillus* strains primarily stems from the production of organic acids and bacteriocins [19,20].

Conventional antibacterial treatments, such as cefazolin and ampicillin, face significant bacterial resistance [21]. Therefore, developing a safe and effective therapeutic agent for oral health is considered vital. In response, probiotics have emerged as a potential defence against antibiotic resistance in severe periodontitis [22]. Effective probiotics must meet certain criteria, including acid and bile resistance, surface adherence, and inhibitory activity against pathogens, all of which are crucial for managing of gingival and periodontal diseases [23]. While various studies have highlighted the inhibitory effects of probiotics on periodontal pathogens, the research specifically focusing on *L. helveticus* in relation to periodontal disease-associated pathogens remains limited. To address this gap, the objective of the present study is to investigate the potential of *L. helveticus* ATCC 15009 strain as a probiotic for promoting oral health, focusing specifically on its antibacterial activity against *P. gingivalis*, a main causative anaerobe bacteria implicated in periodontal disease. Antibacterial susceptibility was assessed using the well diffusion assay, while biofilm biomass was evaluated using crystal violet staining. Cell viability in *P. gingivalis* treated with *L. helveticus* was determined using the LIVE/DEAD assay, employing fluorescence microscopy analysis. Furthermore, ultra-morphological alterations in the treated bacterial cells were examined using FESEM. By investigating this relationship through *in vitro* examination, the study aims to assess the probiotic potential of *L. helveticus* for preventing or mitigating periodontal disease, which ultimately contributes to improved oral health.

## 2. Materials and methods

### 2.1. Bacterial strains and culture conditions

*L. helveticus* ATCC 15009 from the working stock culture was sub-cultured on MRS agar (de Man, Rogosa, Sharpe) for 24 hours at 37°C [24]. Meanwhile, *P. gingivalis* ATCC 33277 was sub-cultured from the working stock culture on supplemented BHI-T blood agar plates (Tryptone Soya agar & Brain Heart Infusion broth), enriched with 5% defibrinated sheep blood, hemin (5.0 mg/mL), menadione (5.0 mg/mL), and cysteine (0.5 g/mL) for 48–72 hours at 37°C under anaerobic conditions, using an anaerobic jar with an Anaerogen gas pack (Thermo Scientific™ Oxoid AnaeroGen 3.5L) [25,26].

Following the initial growth, a loopful of colonies from the agar was transferred into 20 mL MRS broth (de Man, Rogosa, Sharpe) and BHI-T broth for *L. helveticus* and *P. gingivalis*, respectively. The inoculated broth was then incubated for another 24 hours and 48 hours at 37°C under anaerobic conditions, which promoted bacterial multiplication. Subsequently, the bacterial cells were harvested by centrifugation at 10,000 × g for 10 min at 4°C and washed three times with phosphate-buffered saline (PBS). The *L. helveticus* cells were then resuspended in MRS broth, and the bacterial concentration was adjusted spectrophotometrically, which is equivalent to cell densities of approximately 10^7^, 10^8^, and 10^9^ cells/mL based on OD_600_ readings [27–29]. The *P. gingivalis* cells were resuspended in BHI-T broth and was adjusted to optical density (OD_600_) of 0.1 using a UV-VIS spectrophotometer, which is equivalent to approximately 10^8^ colony-forming units per millilitre (cells/mL) [30]. Fresh *P. gingivalis* and *L. helveticus* cultures were Gram-stained for confirmation before being employed in all relevant studies.

### 2.2. Well diffusion assay

The antibacterial activity was tested using the well diffusion method. A sterile cotton swab was employed to evenly distribute 100 µL of *P. gingivalis* bacterial suspension (10^8^ cells/mL) across the surface of the supplemented BHI-T blood agar [31]. Then, the inoculated agar was punctured with a cork borer to create several wells, each with a diameter of 6.0 mm. For the treatment, each well was filled with 30 µL of various concentrations of the *L. helveticus* probiotic suspension (10^7^, 10^8^, and 10^9^ cells/mL). The MRS broth served as the negative control, while 0.12% chlorhexidine gluconate (CHX) was used as the positive control. Subsequently, the agar plates were incubated under anaerobic conditions at 37°C for 72 hours. Following this incubation, the presence or absence of a clear zone around the wells was observed. The diameters of the inhibition zones were measured in millimetres (mm) [32]. Larger inhibition zones indicate greater efficacy, reflecting the ability of the probiotic *L. helveticu*s to inhibit *P. gingivalis.*

### 2.3. Assessment of biofilm activity

Biofilm biomass was determined using crystal violet staining, utilising the broth microdilution method with minor modifications [33]. In a 96-well plate, 100 µL of *P. gingivalis* suspension was added to each well, followed by treatment with 10^9^ cells/mL of probiotic *L. helveticus* suspension at two-fold serial dilutions ranging from 100% to 0.78%. The blank assay consisted of *L. helveticus* suspension at varying concentrations without any bacterial culture, while wells containing the mixture of BHI-T broth and *P. gingivalis* suspension served as negative controls. Additionally, a 0.12% CHX solution was utilised as a positive control. The plates were incubated for 48 hours under anaerobic conditions. Then, the wells were washed three times with sterile PBS. Adherent biofilms were subsequently treated with methanol for 15 minutes, stained with 0.04% (w/v) crystal violet for an additional 15 minutes, and rinsed with deionised water. Finally, 95% ethanol was added to each well, and the optical density (OD) values were measured at 550 nm.

The percentage of biofilm inhibition against *P. gingivalis* at different concentrations of *L. helveticus* suspension was calculated using the following formula [34]:


$$\%\;\text{biofilm\textbackslash{} inhibition}\;=\;[\;1-\;(A_{C}\;/\;A_{0})\times 100$$

where *A*_*C*_ indicates the absorbance of the well with treated *P. gingivalis* (with probiotic) and *A*_*0*_ indicates the absorbance of the control well with untreated *P. gingivalis* (without probiotic).

### 2.4. Live/dead bacterial cell viability assay

In this experiment, a probiotic suspension of *L. helveticus* (10^9^ cells/mL) was added to the wells containing a bacterial suspension of *P. gingivalis* (10^8^ cells/mL) in a 12-well plate, at a volume ratio of 1:1 (v/v) [35]. The samples were incubated at 37°C for two different time points, 24 and 48 hours under anaerobic conditions. Following the incubation period, the samples were centrifuged for 10 minutes at 10,000 × g to pellet the cells, which were then resuspended in supplemented BHI-T broth. Subsequently, 3 µL of the dye mixture (SYTO9 and PI) from the LIVE/DEAD® BacLight™ Kit was added to the bacterial suspension. The dye mixture was then incubated at room temperature in the dark for 15 minutes. Then, an aliquot of the stained cell suspension (5 µL) was dropped onto a glass microscope slide to analyse the morphological changes in both treated and untreated *P. gingivalis* biofilms using a fluorescence microscope (Nikon Eclipse Ti-E Inverted Microscope).

### 2.5. Analysis of morphological alterations in bacterial cells via Field Emission Scanning Electron Microscopy (FESEM)

Briefly, the bacterial suspension of *P. gingivalis* (10^8^ cells/mL) was exposed to the probiotics *L. helveticus* (10^9^ cells/mL) in a 12-well plate, maintaining a 1:1 (v/v) ratio and incubated under anaerobic conditions for 24 hours at 37°C [35]. Following incubation, the suspensions were fixed with 4% glutaraldehyde for 4 hours, rinsed twice in buffer for 10 mins each, and then fixed in 1% osmium tetraoxide for 1 hour. After another rinse with double-distilled water (DDH_2_O) for 10 mins, the samples were dehydrated in increasing concentrations of ethanol (30%, 50%, 70%, 80%, 90%, 95% and 100%) for 15 mins each. The samples were then dehydrated for 15 mins in three different ratios of ethanol to acetone: 3:1, 1:1, and 1:3. The samples were subsequently fixed in pure acetone twice for 20 mins each and dried using critical point drying (CPD) for 1–3 hours. Finally, the samples were mounted on stubs with carbon glue, sputter-coated with gold, and observed using a field emission scanning electron microscope (FESEM).

### 2.6. Statistical analysis

The tests were conducted in triplicate, and the mean ± standard deviation (SD) was calculated. Statistical analyses were carried out using SPSS software (IBM Corp., version 27.0, Armonk, NY, USA). The Shapiro-Wilk test indicated that the data for all experiments did not follow a normal distribution (p < 0.05). To compare data between groups, the non-parametric Kruskal-Wallis test was used, followed by Dunn’s post-hoc test. All analyses were considered statistically significant with p-values < 0.05.

## 3. Results

### 3.1. Effects of *L. helveticus* on the growth inhibition of *P. gingivalis*

**Fig 1:**
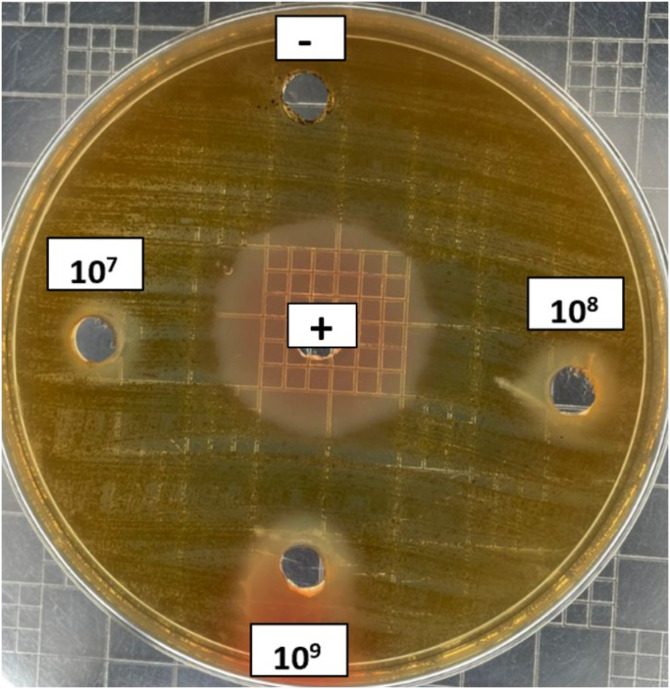
Effect of *L. helveticus* at various concentrations on *P. gingivalis.* A 0.12% chlorhexidine (CHX) solution served as the positive control, and MRS broth was employed as the negative control.

According to the results presented in Table 1 and illustrated in (Fig 1), *L. helveticus* at concentrations of 10^7^ cells/mL, 10^8^ cells/mL and 10^9^ cells/mL effectively inhibited the growth of *P. gingivalis* in a dose-dependent manner. The 10^9^ cells/mL concentration exhibited the largest inhibition zone (14.69 ± 0.78 mm)*,* followed by 10^8^ cells/mL (11.35 ± 0.45 mm) and 10^7^ cells/mL (9.23 ± 0.93 mm). The negative control showed no inhibition. These results indicate that the growth of *P. gingivalis* was most effectively inhibited at 10^9^ cells/mL concentration of *L. helveticus*, leading to its selection for further analysis.

**Table 1:** Measurement of the inhibition zone diameters of *L. helveticus* against *P. gingivalis* in the BHI-T blood agar well diffusion assay.

Treatment (cells/mL)		Zone of inhibition (mm)^a^
***P. gingivalis***
*L. helveticus*	10^7^	9.23 ± 0.93*
	10^8^	11.35 ± 0.45*
	10^9^	14.69 ± 0.78
CHX (0.12%)^b^		23.12 ± 0.62

^a^The zone of inhibition values include a well diameter of 6 mm. A ^b^0.12% CHX solution acted as the positive control. Experiments were conducted in triplicate and values are represented as mean ± standard deviation. Statistical significance was assessed utilising the Kruskal-Wallis test (p-value < 0.05) along with Dunn’s post-hoc analysis, with a significance level set at *p < 0.05 when compared to the control group.

### 3.2. Quantification *P. gingivalis* biofilm biomass inhibition by *L. helveticus* using the crystal violet assay

**Fig 2:**
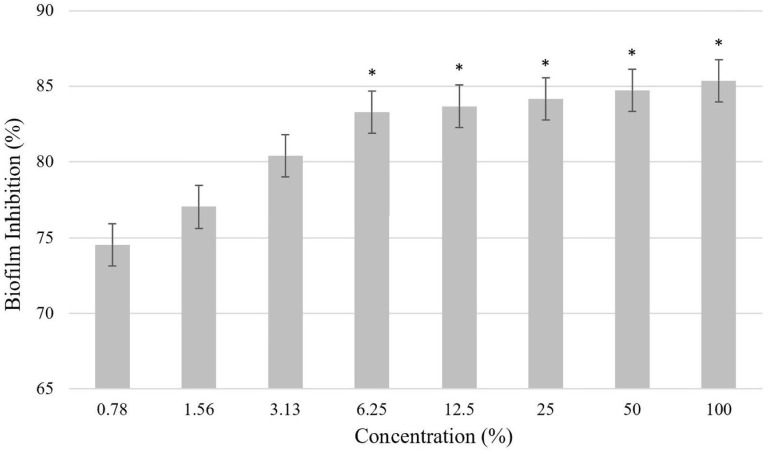
Inhibition of *P. gingivalis* biofilm biomass by *L. helveticus.* The untreated *P. gingivalis* biofilm served as the negative control in this study. Experiments were conducted in triplicate and values are represented as mean ± standard deviation. Statistical significance was assessed utilising the Kruskal-Wallis test (p-value < 0.05) along with Dunn’s post-hoc analysis, with a significance level set at *p < 0.05 when compared to the control group.

The overall biomass of *P. gingivalis* biofilms was quantified using the crystal violet assay, employing two-fold serial dilutions of *L. helveticus* from a stock solution of 10^9^ cells/mL. The results of the biomass analysis are shown in (Fig 2). The *L. helveticus* suspension exhibited significant inhibitory activity against the formation of *P. gingivalis* biofilms. Notably, at the highest concentrations of *L. helveticus*, the treated *P. gingivalis* biofilm displayed a substantial biomass reduction of 85%, as compared to the control group. Hence, these findings demonstrate that *L. helveticus* possesses bacteriostatic properties.

### 3.3. Evaluation of live/dead cell viability *in P.* gingivalis biofilms via fluorescence microscopy

**Fig 3:**
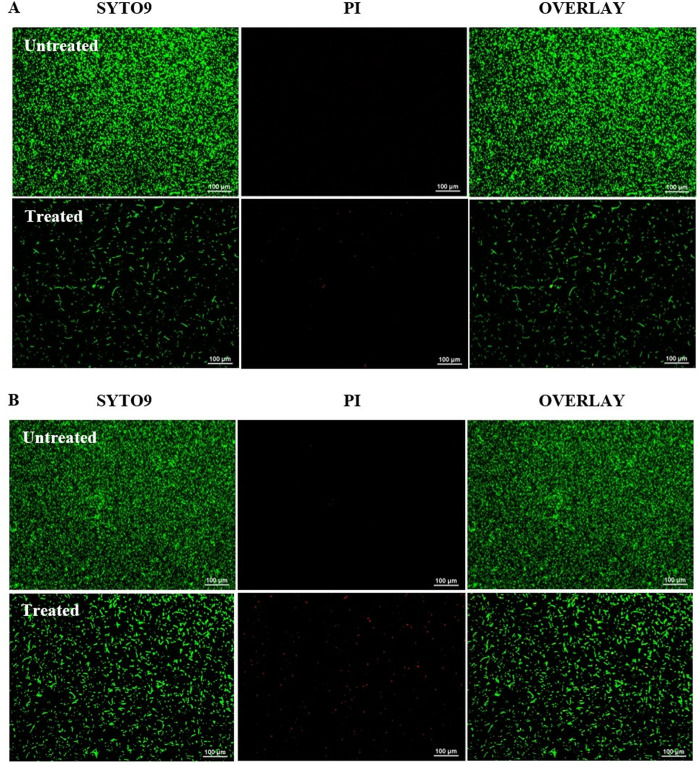
LIVE/DEAD viability staining of untreated and treated *P. gingivalis* biofilm with 10^9^ cells/mL of *L. helveticus*, as revealed under fluorescence microscopy. The biofilm images are viewed after (A) 24 hours and (B) 48 hours of incubation. Viable (live) bacteria cells are green, whereas non-viable (dead) cells are red (Magnification: 10x, scale bar: 100 μm).

Fluorescence microscopy was used to analyse the live/dead cell viability of *P. gingivalis* biofilms after 24 and 48 hours of incubation with *L. helveticus*. Both untreated and treated biofilm samples were stained with the LIVE/DEAD Bacterial Viability Kit, which employs SYTO9 as a green-fluorescent nucleic acid stain and propidium iodide (PI) as a red-fluorescent nucleic acid stain. Cells with intact membranes were stained green (SYTO-9) and classified as viable, whereas those with compromised membranes were stained red (PI) and regarded as dead cells. The overlay images from the assay at 24 hours (Fig 3A), revealed that the untreated *P. gingivalis* biofilm exhibited a highly uniform distribution and dense structure in the absence of *L. helveticus*. Conversely, treatment with *L. helveticus* (10^9^ cells/mL) resulted in significantly dispersed and markedly sparse biofilm structures. The observation indicates that this concentration inhibited *P. gingivalis* biofilm formation, leading to a less dense biofilm.

At 48 hours, a similar trend was observed in the control group, where the biofilm maintained a highly uniform distribution and a dense structure, suggesting increased bacterial growth in the untreated *P. gingivalis* (Fig 3B). Conversely, treated *P. gingivalis* biofilm cells showed an increase in cell numbers during the extended incubation period. Consequently, the structure of the treated *P. gingivalis* biofilm appeared moderately scattered and diffuse. Furthermore, a higher number of dead cells were observed in the treated samples, highlighting the detrimental impact of *L. helveticus* on *P. gingivalis* viability. This evidence collectively demonstrates the significant effects of *L. helveticus* on the morphology and viability of *P. gingivalis* biofilms over time.

### 3.4. The ultra-morphological effects of *L. helveticus* on *P. gingivalis* via FESEM

**Fig 4:**
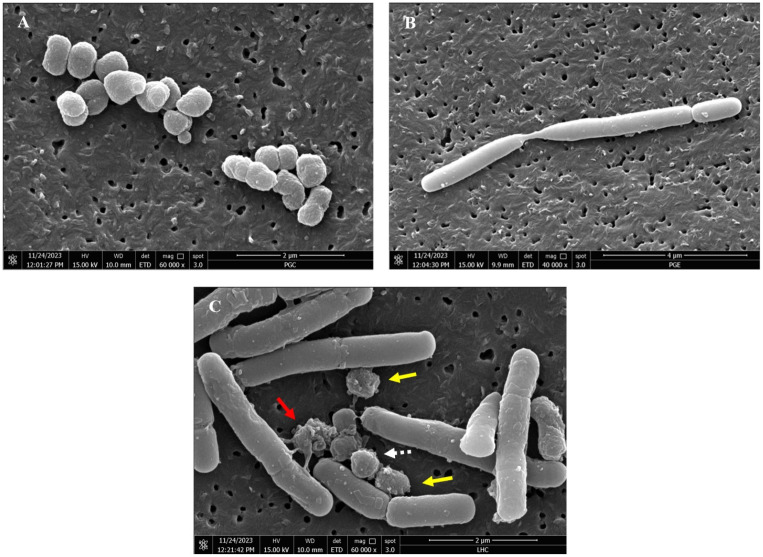
FESEM images of (A) non-treated *P. gingivalis*, (B) non-treated *L. helveticus* and (C) treated *P. gingivalis* with 10^9^ cells/mL concentration of *L. helveticus.* Red arrows: Distortion of cell walls. Yellow arrows: Blisters formation. White dotted arrow: Unaffected *P. gingivalis* cells. Magnification: 60kx (left, below), 40kx (right).

The morphological changes in treated and untreated *P. gingivalis* with *L. helveticus* were assessed using FESEM. The untreated *P. gingivalis* cells were spherical and regular in shape, with smooth surfaces and intact membranes (Fig 4A). In contrast, the viable (negative control) *L. helveticus* cells had smooth surfaces and were rod-shaped with intact structures (Fig 4B). However, after treatment with 10^9^ cells/mL of *L. helveticus*, the *P. gingivalis* cells showed disrupted structures, forming clumps of damaged cells. This observation indicates that *P. gingivalis* exhibited membrane disruption and irregular cell morphology compared to the untreated cells (Fig 4C).

## Discussion

The diseases of periodontitis, gingivitis, and dental caries are caused by microorganisms in the oral cavity. Among these, periodontitis, is a prevalent condition, predominantly associated with Gram-negative bacteria, such as *P. gingivalis*, *Aggregatibacter actinomycetemcomitans (A. actinomycetemcomitans)*, *T. denticola*, and *T. forsythia* [36]. Notably, *P. gingivalis* is recognised as one of the most critical oral pathogens involved in the development of periodontal disease, owing to its various virulence factors that facilitate biofilm formation and contribute to persistent infections in the gingival region [37]. While a substantial literature addresses bacterial biofilms and their effects on periodontal health, further research is needed to fully understand their interactions and explore potential therapeutic strategies. Probiotics, particularly *L. helveticus*, have garnered interest for their oral health benefits. This study investigates the *in vitro* antibacterial effects of probiotic *L. helveticus* ATCC 15009 strain against *P. gingivalis*, providing a preliminary assessment that could guide future therapeutics.

Our study findings revealed a significant inhibitory effect of *L. helveticus* against *P. gingivalis.* The antibacterial activity of *P. gingivalis* was assessed using blood agar, which serves as both a differential and enriched medium. The inclusion of blood in this medium provides essential nutrients for the cultivation of fastidious organisms like *P. gingivalis*. Treatment with the highest concentration of *L. helveticus*, 10^9^ cells/mL demonstrated the largest inhibition zone diameters, indicating that *L. helveticus* displays antibacterial activity towards *P. gingivalis*. Similar to our findings, previous study has reported this strain was among the *Lactobacillus* sp. that has shown inhibitory activity against Gram-negative bacteria in the gut and intestine [38,39]. The data suggested the presence of quorum sensing-promoting molecules (oligopeptides) produced by *L. helveticus* ATCC 15009 are responsible for promoting bacterial communication and therefore enhance the antagonism of *Lactobacillus* sp. against Gram-negative bacteria. Moreover, several preceding studies has found that the inhibitory properties of oral lactobacilli can be attributed to the secretion of various antimicrobial substances or metabolites such as organic acids, hydrogen peroxide, diacetyl, bacteriocins, peptides and other bioactive molecules [40,41]. Specifically, organic acids generated during glucose fermentation, comprising lactic and acetic acid, hinder the growth of strains that are less acid-tolerant [42]. As a result, this may demonstrate the inhibitory activity between *L. helveticus* and *P. gingivalis.*

Once the antibacterial efficiency of the *L. helveticus* strain had been determined, the next stage was to assess its impact on biofilm biomass activity. In natural environments, bacteria primarily exist in structured communities attached to surfaces and surrounded by a self-produced extracellular matrix. When bacterial cells develop biofilm, they produce extracellular polymeric substance (EPS), which protects the biofilm and makes it tough to break down. This provides bacterial cells in biofilm mode with a significant ability to withstand environmental challenges, principally through the protective EPS [43]. These findings suggest that biofilm formation serves as a protective mechanism, redirecting metabolites towards EPS synthesis to provide enhanced protection against stressful conditions by manipulating metabolic processes. The biofilm network creates an anaerobic environment for bacteria embedded within it, allowing them to thrive and secure more resources compared to planktonic cells [44]. Therefore, investigating *L. helveticus* capacity to interrupt biofilm development at various doses is crucial in preventing *P. gingivalis* recolonisation [23].

In this study, the biofilm biomass of *P. gingivalis* exhibited a rapid decrease in the treated samples when compared to the control groups, indicating susceptibility to *L. helveticus*. This noteworthy finding suggests that *L. helveticus* may offer a promising approach for mitigating a significant proportion of *P. gingivalis*-related pathogenicity. These results align closely with various earlier findings which discovered that lactobacilli can disrupt pathogenic bacteria and fungus by fighting for nutrients, co-aggregation, and the generation of antimicrobials such as bacteriocin and hydrogen peroxide, organic acids and influencing the immune system [45]. Moreover, there are few studies showing that lactobacilli can integrate into target biofilms and compete with pathogens for attachment sites in the oral cavity. For example, Vestman et al. (2013) revealed that *L. reuteri* PTA5289 may be absorbed into the oral microbiota for a brief length of time during a 6-week intervention, resulting in a delay in the regrowth of *S. mutans* after oral disinfection with chlorhexidine [46]. Furthermore, James et al. (2016) discovered that a combination of three probiotic strains (*L. helveticus* CBS N116411, *L. plantarum* SD57870, and *S. salivarius* DSM14685) was efficient in both preventing and eliminating *C. albicans* biofilms [47].

The impact of *L. helveticus* were further examined by assessing its action against *P. gingivalis* using a LIVE/DEAD cells viability assay with fluorescence microscopic analysis. The LIVE/DEAD® BacLight^TM^ assay was used to differentiate live and dead cells based on membrane integrity, utilising a dual staining procedure of SYTO 9 and propidium iodide (PI) [48]. Both dyes intercalate with nucleic acids, resulting in an enhanced fluorescent signal. This kit employs SYTO 9 and PI dyes, which have different membrane permeability properties. SYTO 9 can enter all bacterial cell membranes, while PI can only enter cells with disrupted membranes. This allows for distinction between live and dead cells based on the relative green and red fluorescence from SYTO 9 and PI staining [49]. Detection of cell viability using the BacLight Kit offers numerous advantages over colony counting on agar plates, as it provides a rapid and direct quantification of cell death in real time. According to the images obtained, there was a higher level of bacterial cells and a dense biofilm structure of untreated *P. gingivalis* compared to the *P. gingivalis* biofilm treated with 10^9^ cells/mL *L. helveticus.* The exposure of *L. helveticus* to *P. gingivalis* biofilm revealed a clear sparsity and dispersion of the biofilm, indicating a significant reduction in viable cells. These findings support the biofilm assay results, where *P. gingivalis* biofilm formation was substantially inhibited by 10^9^ cells/mL of *L. helveticus*.

In this study, FESEM was utilised to further verify the ultra-morphological effects of *L. helveticus* against *P. gingivalis*. FESEM is an advanced technique used to capture high-resolution images of the microstructure of materials. The FESEM images of the current study revealed morphological changes in *P. gingivalis* cells after treatment with *L. helveticus,* compared to those without treatment, where cells were observed to have regular spherical shapes with smooth surfaces and intact cell membrane. However, not all bacterial cells were affected with *L. helveticus.* Some cells appeared distressed, revealing cell membranes, with blisters forming on the surface, as indicated by the yellow arrows in Fig 4C. Other *P. gingivalis* cells, marked by the white dotted arrow, illustrate that this cell was not affected by *L. helveticus.* In Fig 4C, the red arrow indicates a visible distortion of the cell wall in *P. gingivalis*. This observation suggests a disruption in the bacterium’s structural integrity. Such damage may involve disruption of the cytoplasmic membrane, leading to coagulation of the cell contents and impairing the membrane’s ability to act as a permeability barrier. The binding and antibacterial effect of *L. helveticus* on *P. gingivalis* were evident, warranting further investigation into the specific damage caused by *L. helveticus*-derived peptides. Genetic analysis reported that *P. gingivalis* primarily relies on amino acids for growth, with limited uptake and metabolism of carbohydrates [50]. A previous work on model membranes revealed that bacteriocins could interact with lipid membranes without a specified target antigenic determinant. The contact was most likely caused by an initial electrostatic attraction between the cationic bacteriocins and the anionic lipid membrane, which is a common mechanism found in many antimicrobial peptides [51]. *P. gingivalis* cells have been found to be highly negatively charged at pH values greater than 3 [52]. Matsuzaki et al. (1999) discovered that these positively charged antimicrobial peptides are drawn to the negatively charged components of *P. gingivalis* cells, which gravitate the initial encounter prior to pore development [53]. Similar mechanisms have been found in various cationic antimicrobial peptides, both natural and synthetic [54,55].

In summary, this study highlights important insights into the antibacterial effects of *L. helveticus* against *P. gingivalis*. However, to enhance understanding and address the limitations of these findings, several approaches should be considered. Further studies should focus on elucidating the mechanisms of action of *L. helveticus* and employing models that replicate the oral environment, such as co-culture systems with other oral bacteria. Understanding the interactions among various microbial species, host immune responses, and environmental factors is crucial for evaluating the therapeutic potential of *L. helveticus.* Furthermore, the use of advanced *in vitro* techniques, such as whole genome sequencing and proteomics can provide more in-depth information about how *L. helveticus* influences cell viability and metabolic activity in harmful biofilms. In addition, *ex vivo* and *in vivo* studies should be conducted to validate and strengthen these findings. Addressing these aspects will enhance the development of effective probiotic treatments for improving oral health.

## Conclusion

Succinctly, this study demonstrates that *L. helveticus* ATCC 15009 displayed profound inhibitory effects on the oral pathogen *P. gingivalis,* leading to reduced cell growth and morphological changes in bacteria. Thus, these findings suggest that probiotic therapy could be beneficial in managing periodontal diseases. Moreover, the potent antibacterial effect of *L. helveticus* against *P. gingivalis* emphasizes the importance of further research to identify optimal probiotic strategies that could enhance periodontal health. Understanding how probiotic bacteria can inhibit the growth of pathogenic strains may be crucial in developing preventive measures for periodontitis and optimizing treatment outcomes.

## Supporting information

S1 File The inhibition zone diameters of *L. helveticus* against *P. gingivalis.* (XLSX)

S2 File Effect of *L. helveticus* against *P. gingivalis* biofilm. (XLSX)
